# Inhibition of Osteoarthritis-Related Molecules by Isomucronulatol 7-*O*-β-d-glucoside and Ecliptasaponin A in IL-1β-Stimulated Chondrosarcoma Cell Model

**DOI:** 10.3390/molecules23112807

**Published:** 2018-10-29

**Authors:** Gwan Ui Hong, Jung-Yun Lee, Hanna Kang, Tae Yang Kim, Jae Yeo Park, Eun Young Hong, Youn Ho Shin, Sung Hoon Jung, Hung-Bae Chang, Young Ho Kim, Young-In Kwon, Jai Youl Ro

**Affiliations:** 1 Life & Science Research Center, Hyunsung Vital Co. Ltd., Seoul 07255, Korea; gwanuihong@gmail.com (G.U.H.); legolas018@naver.com (J.Y.P.); h_medical@naver.com (E.Y.H.); dush84@hanmail.net (Y.H.S.); jsh62548@naver.com (S.H.J.); 2 Department of Food and Nutrition, Hannam University, Daejeon 34054, Korea; seembeeks@hanmail.net (J.-Y.L.); hanna9506@hanmail.net (H.K); xodid5606@naver.com (T.Y.K.); 3 Department of Bio Quality Control, Korea Bio Polytechnic, Chungnam 32943, Korea; hbchang@kopo.ac.kr; 4 Department of Pharmacy, Choongnam National University, Daejeon 34134, Korea; yhk@cnu.ac.kr; 5 Department of Pharmacy, Sungkyunkwan University, Suwon 03063, Korea

**Keywords:** chondrosarcoma cells, osteoarthritis, Ryupunghwan (natural product mixture), IL-1β, isomucronulatol 7-*O*-β-d-glucoside, ecliptasaponin A

## Abstract

Osteoarthritis (OA) is the common form of arthritis and is characterized by disability and cartilage degradation. Although natural product extracts have been reported to have anti-osteoarthritic effects, the potential bioactivity of Ryupunghwan (RPH), a traditional Korean medicinal botanical formula that contains *Astragalus membranaceus*, *Turnera diffusa*, *Achyranthes bidentata*, *Angelica gigas*, *Eclipta prostrata*, *Eucommia ulmoides*, and *Ilex paraguariensis*, is not known well. Therefore, the inhibitory effects of single compounds isolated from RPH on the OA-related molecules were investigated using IL-1β-stimulated chondrosarcoma SW1353 (SW1353) cell model. Two bioactive compounds, isomucronulatol 7-*O*-β-d-glucoside (IMG) and ecliptasaponin A (ES) were isolated and purified from RPH using column chromatography, and then the structures were analyzed using ESI-MS, ^1^H-NMR, and ^13^C-NMR spectrum. The expression or amount of matrix metalloproteinase 13 (MMP13), COX1/2, TNF-α, IL-1β or p65 was determined by RT-PCR, Western blot, and enzyme-linked immunosorbent assay (ELISA). RPH pretreatment reduced the expression and amounts of MMP13, and the expression of collagen II, COX1/2, TNF-α, IL-1β or p65, which were increased in IL-1β-stimulated SW1353 cells. IMG reduced the expression of all OA-related molecules, but the observed inhibitory effect was less than that of RPH extract. The other single compound ES showed the reduced expression of all OA-related molecules, and the effect was stronger than that in IMG (approximately 100 fold). Combination pretreatment of both single components remarkably reduced the expression of MMP13, compared to each single component. These synergic effects may provide potential molecular modes of action for the anti-osteoarthritic effects of RPH observed in clinical and animal studies.

## 1. Introduction

The natural product formula popular in Asian countries, Ryupunghwan (RPH), is expected to provide pain relief and reduce inflammation as a health supplement. RPH *contains Astragalus membranaceus*, *Turnera diffusa*, *Achyranthes bidentata*, *Angelica gigas*, *Eclipta prostrata*, *Eucommia ulmoides*, and *Ilex paraguariensis*. 

*Astragalus membranaceus* contains calycosin and is a known Asian medicinal herb traditionally used for the treatment of several diseases, such as hypertension, cirrhosis or cancer therapy through anti-inflammatory and anti-carcinogenic properties, respectively [1]. *Turnera diffusa*, which contains a major constituent arbutin, possesses anti-ulcer activity, which could be attributed to the inhibition of lipid peroxidation, immunomodulatory and anti-oxidant activities [2]. *Achyranthes bidentata* has been shown to protect rat articular chondrocytes against interleukin-1β-induced inflammation and apoptosis in vitro [3]. Thus, it is suggested that it might be a potential drug candidate in the treatment of osteoarthritis (OA) [3]. *Angelica gigas*, known as Chinese Angelica, has been shown to prevent diabetes and liver disease [4]. *Eclipta prostrata* has anti-inflammatory activity in a murine model of asthma [5]. *Eucommia ulmoides* ameliorates arthritis through inhibition of pro-inflammatory cytokines, and through reducing the degradation of cartilage and bone in rat collagen-induced arthritis [6]. *Ilex paraguariensis* can modulate antioxidant defense during perimenopause [7]. Thus, RPH was made by various natural products that have anti-inflammatory effects and reduce the degradation of cartilage, as suggested by folk remedies or laboratory experiments.

OA, which affects most mammalian populations, is the most common form of arthritis and is one of the leading causes of disability worldwide. In humans, 9.6% of men and 18% of women over the age of 60 years have symptomatic OA [8]. Ageing, obesity, gender, increased biomechanical loading of joints, and genetics or low-grade systemic inflammation have been known as risk factors for OA [9,10]. Although the mechanistic details of OA pathogenesis remain to be elucidated, the cartilage degradation and inflammation is the most predominant pathological feature that inevitably leads to joint dysfunction [11,12]. The articular cartilage is composed of water and extracellular matrix (ECM), which mainly composed of Type II collagen, aggrecan, proteoglycans and other collagen subtypes [10]. Thus, the cartilage homeostasis is maintained by the balance between ECM synthesis and degradation [12]. Disturbances of this balance are one of the main characteristics of OA cartilage, and the restoration of balance is the key factor for cartilage regeneration in OA. Inflammation in early OA is caused through inflammatory mediators, particularly TNF-α, IL-1β [13], and prostaglandin E2 (PGE_2_), which is produced through degradation of arachidonic acid by COX-2 [14].

Recently, NSAIDs, which are used in the clinic for OA therapy, target the cyclooxygenase (COX) enzymes, key enzymes in the synthesis of prostaglandins produced in sites of tissue damage or infection, and inhibit both enzymes COX-1 and COX-2. Inhibition of COX-1 often results in gastrointestinal adverse effects, and the COX-2 is known as a regulator of inflammation [15]. The selective COX-2 inhibitor like celecoxib is used effectively for the pain relief and inflammation of OA [16,17]. Recently, new biological agents, such as bone morphogenetic protein-7 and growth factors etc., are known to stimulate chondrogenesis, inhibit matrix degradation and reduce inflammation [18]. However, in addition to an increased side effect such as gastrointestinal, cardiovascular adverse events and complications [19], these agents have failed to block the progression of OA [18,20]. Therefore, safer and better-tolerated solutions for the treatment of OA need to be developed. 

Based on the findings above, this study investigates for the first time whether the RPH has inhibitory effects on the OA-related molecules in IL-1β-stimulated SW1353 cells. After confirmation the inhibitory effects of OA, this study also aimed to isolate single functional components contained in RPH and to investigate whether the single components have osteoarthritic health benefits. The data suggest that RPH may contribute to the development of a health functional food and valuable information for OA. 

## 2. Results

### 2.1. Effects of Natural Product Mixture (Ryupunghwan, RPH) on the Expression of Osteoarthritis (OA)-Related Molecules or Cyclooxygenase (COX) in IL-1β-Stimulated Chondrosarcoma SW1353 Cells

**Figure 1:**
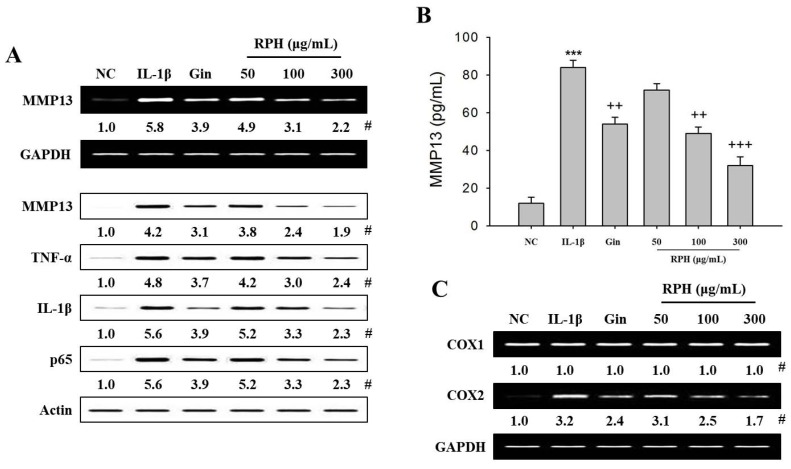
Effects of natural product mixture (Ryupunghwan (RPH)) on the expression or amount of osteoarthritis (OA)-related molecules or cyclooxygenase (COX) in IL-1β-stimulated chondrosarcoma SW1353 cells (SW1353 cells). SW1353 cells (1 × 10^6^ cells) were stimulated with 20 μg/mL IL-1β for 24 h. Natural product mixture (RPH, 50, 100, 300 μg/mL) or ginsenoside (Gin, 300 mg/mL) was pretreated for 1 h before IL-1β. The expression of MMP13, COX-1, COX-2, TNF-α or IκBα was determined in protein or mRNA extracts isolated from the cell lysates using Reverse Transcription-Polymerase Chain Reaction (RT-PCR) or Western blot, respectively. Amount of MMP13 was determined in the cell supernatant isolated from IL-1β-stimulated cells using enzyme-linked immunosorbent assay (ELISA) kit. (**A**) Expression of MMP13 and OA-related molecules (TNF-α, IL-1β, or IκBα). (**B**) Amount of MMP13. (**C**) Expression of COX1/2. #, Numbers below band images show the mean values (*n* = 4) obtained from the ratio of band density of each group versus those of the control and loading control GAPDH or actin. Results for ELISA assay represent the mean ± SEM (*n* = 4) obtained from four independent experiments performed in triplicates. NC, negative control; IL-1β (interleukin 1 beta), IL-1β-stimulated SW1353 cells; Gin, ginsenoside. ***, *p* < 0.001 versus the NC. ^++^, *p* < 0.01; ^+++^, *p* < 0.001 versus the IL-1β stimulation.

The OA joints when stimulated by IL-1β, produce high levels of a variety of MMPs, particularly MMP13 [21,22]. Thus, we examined whether RPH has any influence on the expression of various molecules (MMP13, TNF-α, IL-1β, IκBα, and COX1/2) related to cartilage degradation and inflammation of OA in IL-1β-stimulated SW1353 cells. Expression of MMP13 mRNA and protein, and the amount of MMP13 secreted was increased in IL-1β-stimulated SW1353 cells versus negative control (NC). RPH pretreatment (50, 100, 300 μg/mL) reduced the expression of MMP13 in a dose-dependent manner (Figure 1A). 

It has been reported that TNF-α is pro-inflammatory cytokine involved in IL-1β-induced chondrocytes or OA [13,23], that NF-κB is related to the production of inflammatory molecules in chondrocytes [24,25]. Thus, we examined whether RPH affects the expression of OA related-molecules in IL-1β-stimulated SW1353 cells. The expression of TNF-α, IL-1β, and NF- kB subunit p65 was significantly increased in IL-1β-stimulated SW1353 cells versus NC. RPH pretreatment inhibited these molecules, which are typically increased in IL-1β-stimulated SW1353 cells, in the dose-dependent manner (Figure 1A), but it increased expression of IκBα. Pretreatment of 300 μg/mL (high dose of RPH) of ginsenoside, which is used as one of natural positive control of natural product mixture (RPH), inhibited the expression of all OA-related molecules (MMP13, TNF-ɑ, IL-1β) which were observed in RPH as well as amount of MMP13, but it increased expression of IκBɑ. However, its efficacy was less than those in 100 μg/mL of RPH (middle dose). 

We examined whether RPH has an effect on the expression of COX1/2, which are known as a regulator of inflammation [21]. The expression of COX-2 was significantly increased in IL-1β-stimulated SW1353 cells versus NC. RPH pretreatment inhibited the increased expression of this enzyme in a dose-dependent manner. Pretreatment of 300 μg/mL Gin inhibited COX-2 expression less than that in 100 μg/mL RPH (middle dose). However, constitutive COX-1 did not show any change in this experimental groups pretreated with RPH or Gin (Figure 1C). 

### 2.2. Purification and Identification of Bioactive Ingredients from RPH

**Figure 2:**
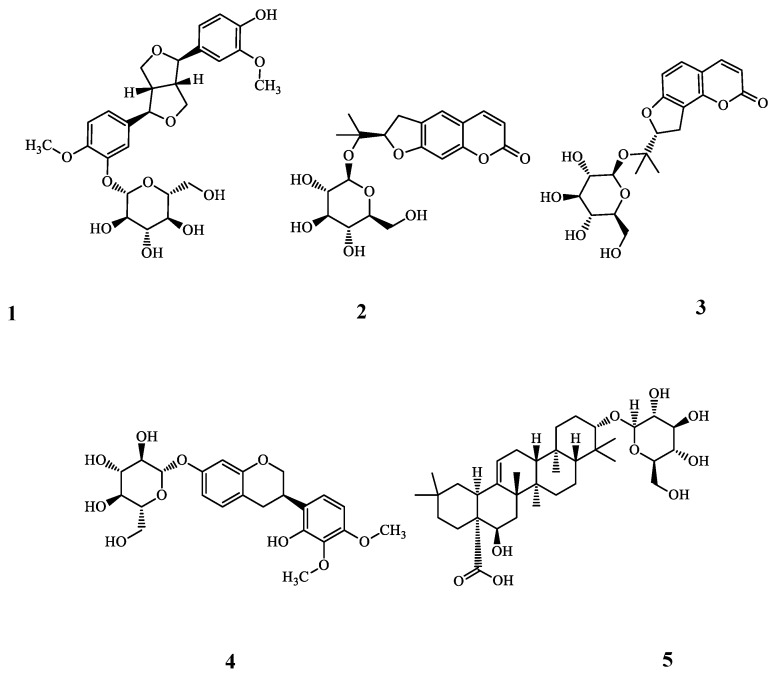
Chemical structures of compounds isolated from RPH. RPH (1.5 kg) was fractioned using various organic chemicals and columns as described in “Materials and Methods”. Finally, five single components were identified from RPH, and then structure of each component was analyzed using ESI-MS, ^1^H-NMR, and ^13^C-NMR spectrum. Inhibitory effects of each fraction identified from organic chemicals were determined in the expression of MMP13 (data not shown). Structures of (**1**), (**2**), (**3**), (**4**), or (**5**) indicate (−)-pinoresinol 4-*O*-β-d-glucopyranoside, (−)-marmesinin, columbianetin β-d-glucopyranoside, isomucronulatol 7-*O*-β-d-glucoside (IMG) and ecliptasaponin A (ES), respectively.

To investigate single component, which has anti-OA efficacy contained in RPH, we used a variety of methods. Ryupunghwan (RPH) as a natural product mixture and its contents are described in the materials and methods section. RPH was suspended in distilled water under ultrasonic agitation at 90 Hz and 40 °C and successively partitioned with ethyl acetate and n-BuOH to afford ethyl acetate and n-BuOH, and water fractions. n-BuOH extracts of RPH showed potent inhibitory effects on the expression of MMP13 (data not shown). n-BuOH fraction was subjected to silica gel and C-18 column chromatography, and five compounds (**1**–**5**) were isolated (Figure 2). The spectroscopic data (ESI-MS, ^1^H-NMR, and ^13^C-NMR spectra) and comparisons with previous data confirmed that these structures were (−)-pinoresinol 4-*O*-β-d-glucopyranoside (**1**) [26], (−)-marmesinin (**2**) [27], columbianetin β-d-glucopyranoside (**3**) [28], isomucronulatol 7-*O*-β-d-glucoside (**4**) [29], and ecliptasaponin A (**5**) [30] (Figure 2, Supplementary Materials Figures S1–S15).

### 2.3. Effects of Single Component IMG and ES Isolated from Natural Product Mixture (RPH) on the Expression of OA-Related Molecules in IL-1β-Stimulated SW1353 Cells

**Figure 3:**
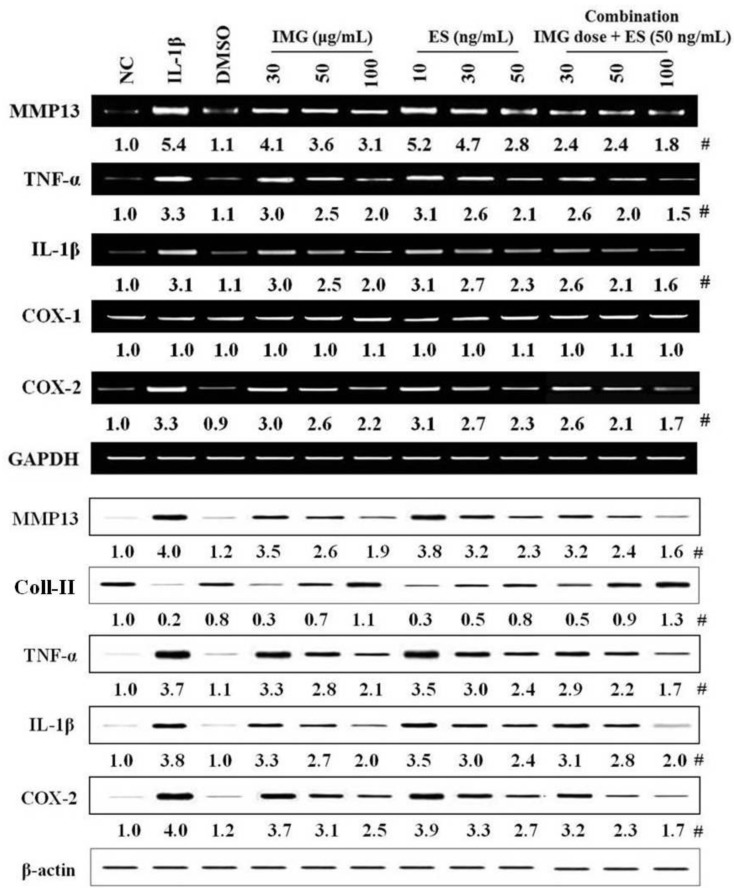
Effects of single component IMG and ES isolated from natural product mixture (RPH) on the expression of OA-related molecules in IL-1β-stimulated SW1353 cells. SW1353 cells (1 × 10^6^ cells) were stimulated with 20 μg/mL IL-1β for 24 h. IMG (isomucronulatol 7-β-*O*-glucoside, 30, 50 or 100 μg/mL), ES (ecliptasaponin A, 10, 30 or 50 ng/mL), or combination (each dose of IMG + 50 ng/mL ES) was pretreated at 1 h before IL-1β stimulation. The expression of OA-related molecules including collagen type II was determined in mRNA extracts isolated the cell lysates using RT-PCR, and Western blot, respectively. (Upper panel) The mRNA expression of OA-related molecules (MMP13, TNF-α, IL-1β, COX1/2). (Lower panel) The protein expression of OA-related molecules (MMP13, Collagen type II, TNF-α, IL-1β, and COX-2). #, Numbers below band images are the mean values (*n* = 4) obtained from as described in the Figure 1 legend. NC, negative control; IL-1β (interleukin 1 beta), IL-1β-stimulated SW1353 cells; DMSO (0.3%), control for the solvent of a single component; Coll-II, collagen type II.

Five single compounds were isolated from RPH, and to be the responsible bioactive compounds. Two substances (isomucronulatol 7-*O*-β-d-glucoside (IMG) and ecliptasaponin A (ES)) among five substances showed the suppression of molecules, MMP13, collagen type II, TNF-α, IL-1β, and COX-2, typically related to OA. One of single components IMG (30, 50, 100 μg/mL) suppressed the expression of MMP13, collagen type II, TNF-α, IL-1β, and COX-2 related to OA in IL-1β-stimulated SW1353 cells in a dose-dependent manner (Figure 3). The other single component ES (10, 30, 50 ng/mL) remarkably reduced the expression of all molecules related to OA versus IL-1β-stimulated SW1353 cells. IMG showed less potency than that in RPH. On the other hand, ES has stronger potency than that of RPH or IMG, but toxicity in terms of cell lysis was observed in the high dose above 70 ng/mL (data not shown). However, the potency of ES was stronger by approximately 1000 times than IMG. Therefore, we tried the combination pretreatment of both single functional components. The combination pretreatment of IMG (30, 50, 100 μg/mL) with ES, which fixed on 50 ng/mL of dose, remarkably suppressed expression of all OA-related molecules by approximately 48~67% (100 μg/mL of IMG plus 50 ng/mL of ES) in IL-1β-stimulated SW1353 cells (Figure 3). 

## 3. Discussion

We demonstrate that natural product mixture Ryupunghwan (RPH) reduces the expression or secretion of OA-related molecules such as MMP13, TNF-α, p65 and COX1/2, and that two compounds (Isomucronulatol 7-*O*-β-d-glucoside, IMG; Ecliptasaponin A, ES) among five single compounds, which are isolated and purified from RPH and then their structures are analyzed, have functional activities. A single functional compound IMG is a known compound present in *Astragalus propinquus* [1,31] that has the highest percentage in RPH, but its functional activity for OA has not been evaluated yet. *Astragalus propinquus* contains calycosin-7-*O*-β-d-glucopyranoside as an index material, which had anti-inflammation and anti-osteoarthritis properties [32,33]. The functional activity in OA for ES was also not clarified yet, although ES is found in *Eclipta prostrata* L. and is reported as an index material, which exhibited anti-inflammatory activity in an allergic asthma model in mice [5]. There is only one report that ES shows a protective effect against lung tissue inflammation in the bleomycin-induced pulmonary fibrosis via reducing the oxidative stress [34]. 

The pathological feature for OA, which is caused by various risk factors such as ageing and low-grade systemic inflammation etc., is characterized by cartilage degradation and inflammation of joint [11,13]. Increased MMP13 expression is related to cartilage degradation [21,22]. Inflammatory cytokines TNF-α and IL-1β are associated with inflammation of joint in early OA [13,14]. NF-κB subunit p65 as a transcriptional factor causes the production of inflammatory cytokines [14,24,25]. The articular cartilage is mainly composed of collagen type II, aggrecan and proteoglycans subtypes [10]. Prostaglandins (PGE_2_), which recruited/activated inflammatory cells into synovia, are produced via COX2 [14]. Thus, our data suggest that RPH may reduce cartilage degradation and inflammation in OA through inhibiting the expression of MMP13, collagen type II, COX2 and inflammatory cytokines, as demonstrated by the data showing that RPH reduced expression of all OA-related molecules investigated. The data can also be inferred that RPH may induce the regeneration of cartilage via reducing the expression of MMP13 and collagen type II in OA. 

RPH has no effect on COX1 expression, which is known as an enzyme to regulate the secretion of gastric acid in gastric intestinal tract [35]. This phenomenon implies that RPH is natural product mixture that could be used for OA management and have less toxicity because it has no influence on COX1 expression.

Also, our observations suggest that single component IMG and ES isolated in RPH may inhibit the expression of MMP13 and COX2, which is typically associated with the degeneration of cartilage in osteoarthritis, and inflammatory cytokines, and expression of COX2 which is known as a regulator of inflammation. 

In conclusion, these in vitro results support that single functional compounds IMG and ES have significantly anti-osteoarthritic effect in IL-1β-stimulated chondrosarcoma SW1353 cells. The data suggest that RPH containing IMG and ES with less side-effects, which have anti-oxidative and anti-inflammatory properties associated with osteoarthritis, may have a potential as a health functional food supplement for osteoarthritis, with less side-effects and should be further evaluated in animal and clinical models.

## 4. Materials and Methods

### 4.1. Materials

Ryupunghwan (RPH), a natural product mixture was donated by Hyunsung Vital Co. Ltd. (Seoul, Korea). RPH contains *Astragalus propinquus* (31%), *Turnera diffusa* (14%), *Achyranthes bidentate* (14%), *Angelica sinensis* (14%), *Eclipta prostrata* (12%), *Eucommia ulmoides* (8%), and *Ilex paraguariensis* (7%). These plants were extracted using hot water (90 °C) for 24 h and evaporated by Liquefied extractor (Hyunsung Vital Co. Ltd., Seoul, Korea) to yield a powder of RPH.

### 4.2. Purification and Identification of Bioactive Ingredients

The Ryupunghwan (RPH, 1.5 kg) was suspended in distilled water (4.0 L) under ultrasonic agitation at 90 Hz and 40 °C and successively partitioned with ethyl acetate and n-BuOH to afford ethyl acetate (96.0 g, A) and n-BuOH (80.0 g, B), and water fractions.

The n-BuOH fraction (B) showed potent inhibitory effects on the expression of osteoarthritis (OA)-related molecules or cyclooxygenase (COX), this fraction was chosen for subsequent studies. The n-BuOH fraction was separated using a silica gel column with a gradient solvent mixture of CHCl_3_-MeOH-H_2_O (10:1:0, 6:1:0.1, 4:1:0.1, 2:1:0.1, and 1:1:0.1) to afford six subfractions (B-1 to B-6). Next, subfraction B-1 (12.0 g) was subjected to silica gel CC and was eluted with a solvent mixture of CHCl_3_-acetone (10:1, and 7:1) and CHCl3-MeOH-H_2_O (7:1:0.1, 5:1:0.1) to afford four subfractions (B-1.1 to B-1.4). Further purification of subfraction B-1.1 (1.4 g) via YMC RP-C18 silica gel column using mixtures of MeOH–H_2_O (1:1, 1.5:1, and 2:1) yielded compounds **1** (80.0 mg), **2** (78.0 mg), and **3** (90.0 mg). When the same steps were repeated as above, compounds **4** (20.0 mg), and **5** (60.0 mg) were also obtained by purifying subfraction B-1.2 (2.0 g) on YMC RP-C18 silica gel using mixtures of MeOH–H_2_O (1.5:1, and 2:1). 

### 4.3. Human Chondrosarcoma SW1353 Cells Culture Conditions

Human chondrosarcoma cells (SW1353 cells) were obtained from American Type Culture Collection (ATCC; No. HTB-94, Manassas, VA, USA). The cells were grown in Leibovitz’s L-15 medium (Gibco, Grand Island, NY, USA) supplemented with 1% l-glutamine (Sigma-Aldrich, St. Louis, MO, USA), 1% antibiotic penicillin/streptomycin solution (Sigma-Aldrich, St. Louis, MO, USA), and 10% fetal bovine serum (HyClone, Logan, UT, USA). The cells were maintained at 37 °C in a humidified atmosphere without CO_2_, and the media was replaced every 3 days [36].

### 4.4. Cell Stimulation and Treatment

After serum starvation for 24 h, the cells (1 × 10^6^ cells) were stimulated with 20 μg/mL recombinant human interleukin-1 beta (IL-1β; PeproTech, Rocky Hill, NJ, USA), and then incubated for 24 h [37]. The cells were centrifuged (470× *g*, 3 min) to separate the supernatants and cells. The supernatants were used to measure the amount of MMP13, and the cells used to determine the expression of all molecules related to OA. Optimal concentrations for IL-1β stimulation, RPH, single component or Gin (20 μg/mL) were yielded in the preliminary experiments (data not shown). Dimethyl sulfoxide (DMSO; 0.3%), ginsenoside (300 μg/mL), natural product mixture (RPH; 50, 100 or 300 μg/mL), or isomucronulatol 7-β-*O*-glucoside (SG; 30, 50 or 100 μg/mL) and ecliptasaponin A (ES; 10, 30 or 50 ng/mL) as a single component separated from natural mixture were pretreated at 1h before IL-1β stimulation. 

### 4.5. Reverse Transcription-Polymerase Chain Reaction (RT-PCR)

Total mRNA was extracted from the SW1353 cells (1 × 10^6^ cells) using TRIzol reagent (Invitrogen, Life Technologies Ltd., UK). RT-PCR was performed in a final volume of 20 μL using a High-capacity cDNA Reverse Transcription kit (Applied Biosystems, Foster City, CA, USA) and G-taq kit (Cosmogenetech, Seoul, Korea) in an automated thermal cycler (Bio-Rad, Laboratories, CA, USA). PCR assays were performed for 35 cycles. Each cycle consisted of following steps: denaturation at 94 °C for 30 s, annealing at 56 °C for 45 s, and extension at 72 °C for 1 min. The result was expressed as a ratio of GAPDH mRNA. PCR products were analyzed using 1% agarose gel and visualized under a UV light after staining with stay safe nucleic acid gel stain (Real Biotech Corporation, Banqiao, Taiwan) [38].

The primer sequences used were as follows: MMP13 sense, 5′-TCC CAG GAA TTG GTG ATA AAG TAG A-3′; MMP13 anti-sense, 5′-CTG GCA TGA CGC GAA CAA TA-3′; TNF-α sense, 5′-TCT ACT CCC AGG TCC TCT TC-3′; TNF-α anti-sense, 5′-AAG TAG ACC TGC CCA GAC TC-3′; IL-1β sense, 5′-CTT TGA AGC TGA TGG CCC TAA A-3′; IL-1β anti-sense, AGT GGT GGT CGG AGA TTC GTA-3′; COX1 sense, 5′-CGC GGA TCC ACC ATG AGC CGG-3′; COX1 anti-sense, 5′-TGC TTT CAA GCT TCT CAG-3′; COX2 sense, 5′-TTG CGG CCG CCA CCA TGG TCG -3′; COX2 anti-sense, 5′-GCT CTA GAG ACT TCT ACA GTT CAG-3′; GAPDH sense, 5′-AAC TTT GGC ATT GTG GAA GG-3′; GAPDH anti-sense, 5′-ACA CAT TGG GGG TAG GAA CA-3′.

### 4.6. Preparation of Nuclear Extracts

The SW1353 cells (1 × 10^6^ cells) harvested from IL-1β-stimulated cells were suspended in a cytoplasmic extraction buffer [10 mM HEPES, 60 mM KCl, 1 mM EDTA, 0.075% (*v*/*v*) NP40, 1mM DTT, 1 mM PMSF and 2.5 ug/mL each of aprotinin, leupeptin, and pepstatin), adjusted to pH 7.6], and allowed to incubate on ice for 15 min. After removing the cytoplasmic extract, the cell pellets were washed with cytoplasmic extraction buffer without NP40. After spin down, and the cell pellet was treated with the nuclear extraction buffer [20 mM Tris Cl, 420 mM NaCl, 1.5 mM MgCl_2_, 0.2 mM EDTA, 0.5 mM PMSF and 25% (*v*/*v*) glycerol, adjusted to pH 8.0]. The final salt concentration was adjusted to 400 mM with NaCl. The extracts were incubated on ice for 30 min, and then the supernatants were collected [39]. Nuclear extracts (50 μg) in supernatants were used to measure for expression p65 using Western blot.

### 4.7. Western Blot Analysis

The SW1353 cells (1 × 10^6^ cells) harvested from IL-1β-stimulated cells were suspended in a low-salt lysis buffer [10 mM HEPES (pH 7.9), 10 mM KCl, 0.1 mM EDTA, 0.1 mM EGTA, 1 mM DTT, 0.5 mM PMSF, 2 μg/mL aprotinin, 2 μg/mL leupeptin] and allowed to swell on ice for 30 min. The cells were then homogenized using a Polytron homogenizer (Kinematica, Lucern, Switzerland). After centrifugation, supernatants obtained from cells extracts were analyzed by 10% SDS-polyacrylamide gel electrophoresis and electrophoretically transferred to nitrocellulose membranes (Amersham Biosciences, Piscataway, NJ, USA). The membranes were washed with PBS containing 0.1% Tween 20 (PBST) and then blocked for 1 h in PBST containing 5% skim milk. After washing the membranes with PBST, they were treated with primary Abs against actin, MMP13, TNF-α, IL-1β, COX-2, or P65 (Cell Signaling Technology, Bevery, MA, USA), or collagen type II (Santa Cruz, CA, USA) diluted with PBST (1:1000). Membranes were washed with PBST and treated with horseradish peroxidase (HRP)-conjugated goat anti-mouse or HRP-conjugated goat anti-rabbit IgG (diluted to 1:5000~1:10,000) (Bethyl Laboratories, Montgomery, TX, USA) in PBST for 1 h. After washing, the protein bands were visualized by Enhanced Chemi-Luminescence using a chemiluminometer (ECL; Amersham Biosciences, Piscataway, NJ, USA) [40].

### 4.8. MMP13 Amount Assay

The amount of MMP13 secreted in supernatants (100 μL) isolated from media of cells stimulated with IL-1β was determined using MMP13 ELISA kit (Abcam, Cambridge, UK). Briefly, 100 μL standard solution or samples were added in 96 well plates coated with specific human MMP13 and then incubated for an overnight on a microplate shaker. Each well was washed 3 times with 300 μL of diluted wash buffer, and then 150 μL of HRP-conjugated streptavidin (Abcam, Cambridge, UK) was added and incubated for 1 h. The wells are again washed, a TMB substrate solution was added to the wells, and the color was developed in proportion to a number of MMP13 bound. The plates were read at 450 nm using microplate spectrophotometer (Molecular Devices, Sunnyvale, CA, USA). The amounts of MMP13 were calculated using the standard curves generated by specific MMP13 standards. The results were expressed in ng/mL (1 × 10^6^ cells). The lowest detection limit for MMP13 was 8.23 pg/mL [41].

### 4.9. Statistical Analysis

Experimental data are shown as means ± SEM (*n* = 8). The unpaired Student’s *t*-test was used to compare the two groups. Multiple group comparisons were performed using two-way analysis of variance (ANOVA) followed by Scheffe’s post hoc test, using the SPSS 11 software (SPSS Inc., Chicago, IL, USA). *p* values < 0.05 were considered to indicate statistical significance. The densitometry analysis of Western blots and RP-PCR were performed with Quantity One (version 4.6.3; Bio-Rad, Hercules, CA, USA) and are indicated as means ± SEM (*n* = 4) obtained from the ratio of each band density versus those in the control and loading control of four independent experiments using the densitometer.

## Supplementary Materials

The following are available online. Figure S1: ^1^H-NMR of (−)-pinoresinol 4-*O*-β-d-glucopyranoside (1); Figure S2: ^13^C-NMR of (−)-pinoresinol 4-*O*-β-d-glucopyranoside (**1**); Figure S3: ESI-MS spectra of (−)-pinoresinol 4-*O*-β-d-glucopyranoside (**1**); Figure S4: ^1^H-NMR spectra of (−)-marmesinin (**2**); Figure S5: ^13^C-NMR spectra of (−)-marmesinin (**2**); Figure S6: ESI-MS spectra of (−)-marmesinin (**2**); Figure S7: ^1^H-NMR spectra of columbianetin β-d-glucopyranoside (**3**); Figure S8: ^13^C-NMR spectra of columbianetin β-d-glucopyranoside (**3**); Figure S9: ESI-MS spectra of columbianetin β-d-glucopyranoside (**3**); Figure S10: ^1^H-NMR spectra of isomucronulatol 7-*O*-β-d-glucoside (**4**); Figure S11: ^13^C-NMR spectra of isomucronulatol 7-*O*-β-d-glucoside (**4**); Figure S12: ESI-MS spectra of isomucronulatol 7-*O*-β-d-glucoside (**4**); Figure S13: ^1^H-NMR spectra of ecliptasaponin A (**5**); Figure S14: ^13^C-NMR spectra of ecliptasaponin A (**5**); Figure S15: ESI-MS spectra of ecliptasaponin A (**5**).
