# Supplementary material for: Inhibition of Osteoarthritis-Related Molecules by Isomucronulatol 7-O-β-d-glucoside and Ecliptasaponin A in IL-1β-Stimulated Chondrosarcoma Cell Model
Source: Molecules. 2018 Oct 29;23(11):2807. doi: 10.3390/molecules23112807 (PMC6278319; doi:10.3390/molecules23112807)
Supplement: Supplementary file 1 [file molecules-23-02807-s001.pdf]

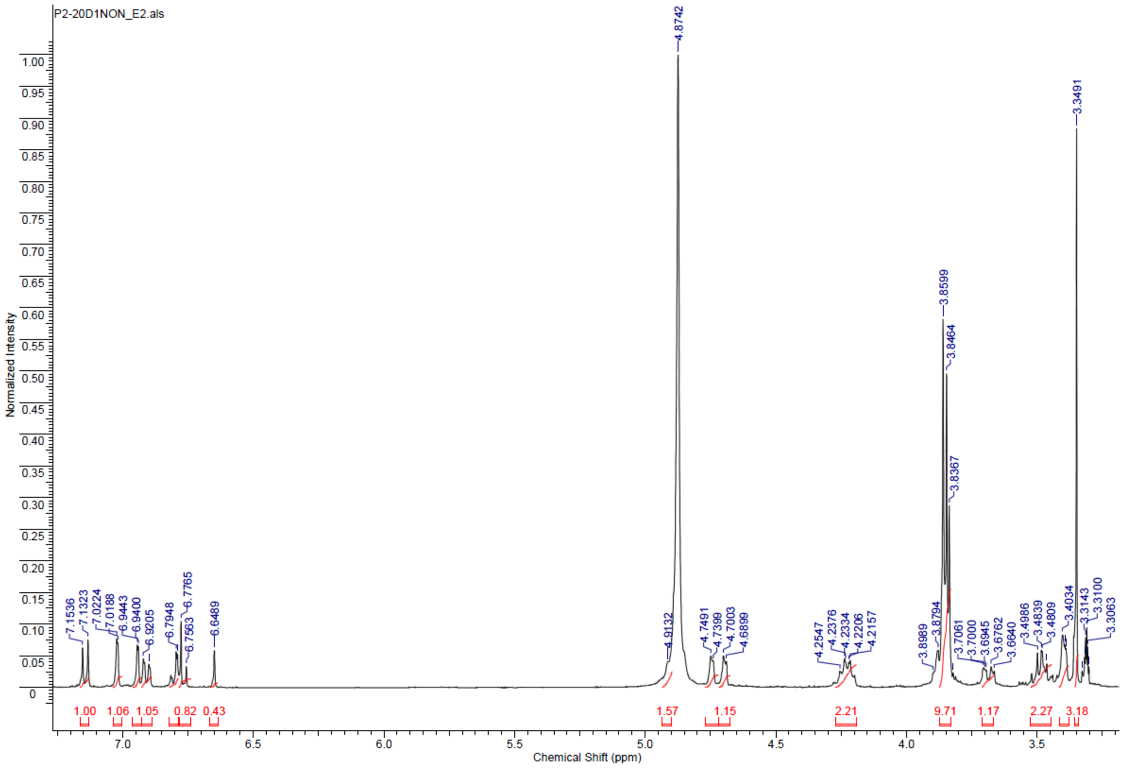

Figure S1. ^{1}H-NMR of (-)-pinoresinol 4-*O*-β-D-glucopyranoside (**1**).

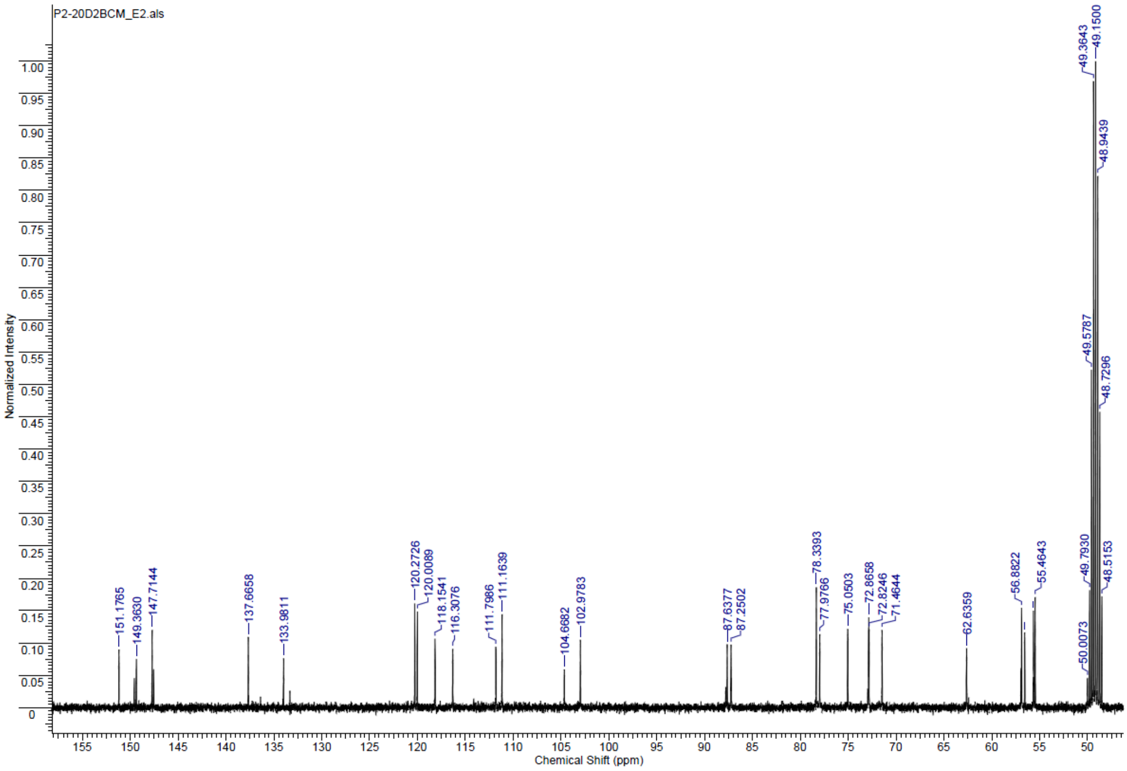

Figure S2. ^{13}C-NMR of (-)-pinoresinol 4-*O*-β-D-glucopyranoside (**1**).

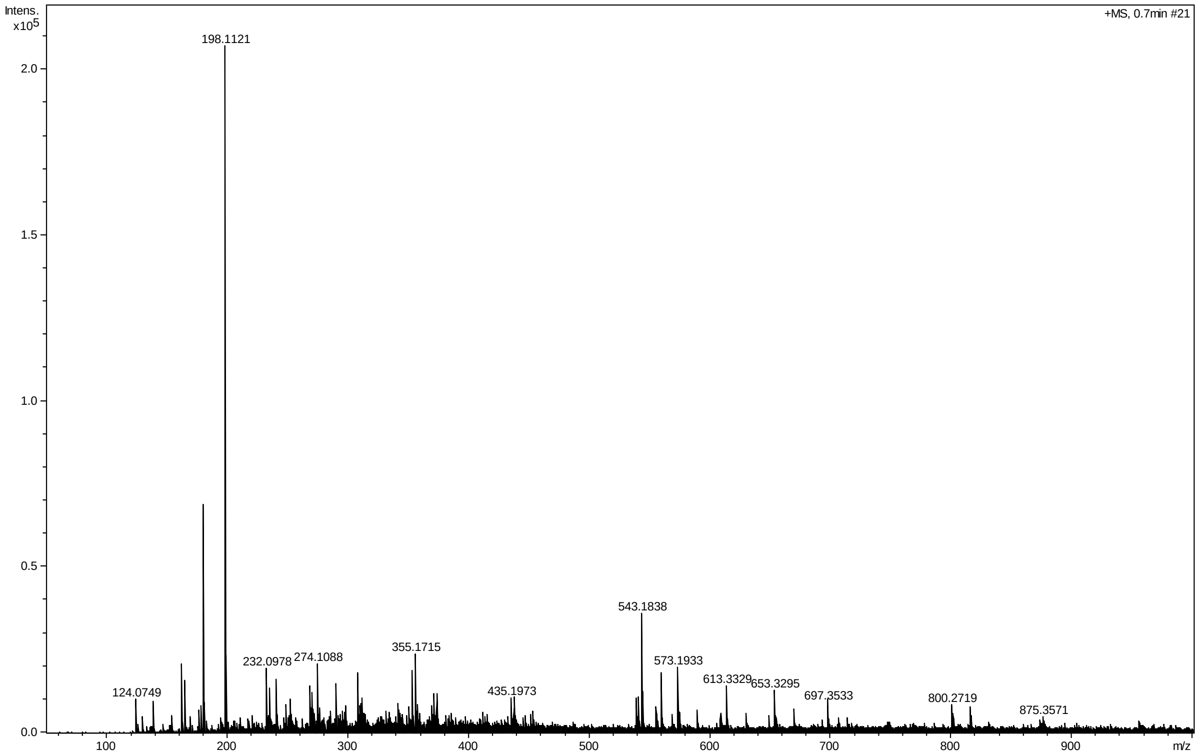

Figure S3. ESI-MS spectra of (-)-pinoresinol 4-*O*-β-D-glucopyranoside (**1**).

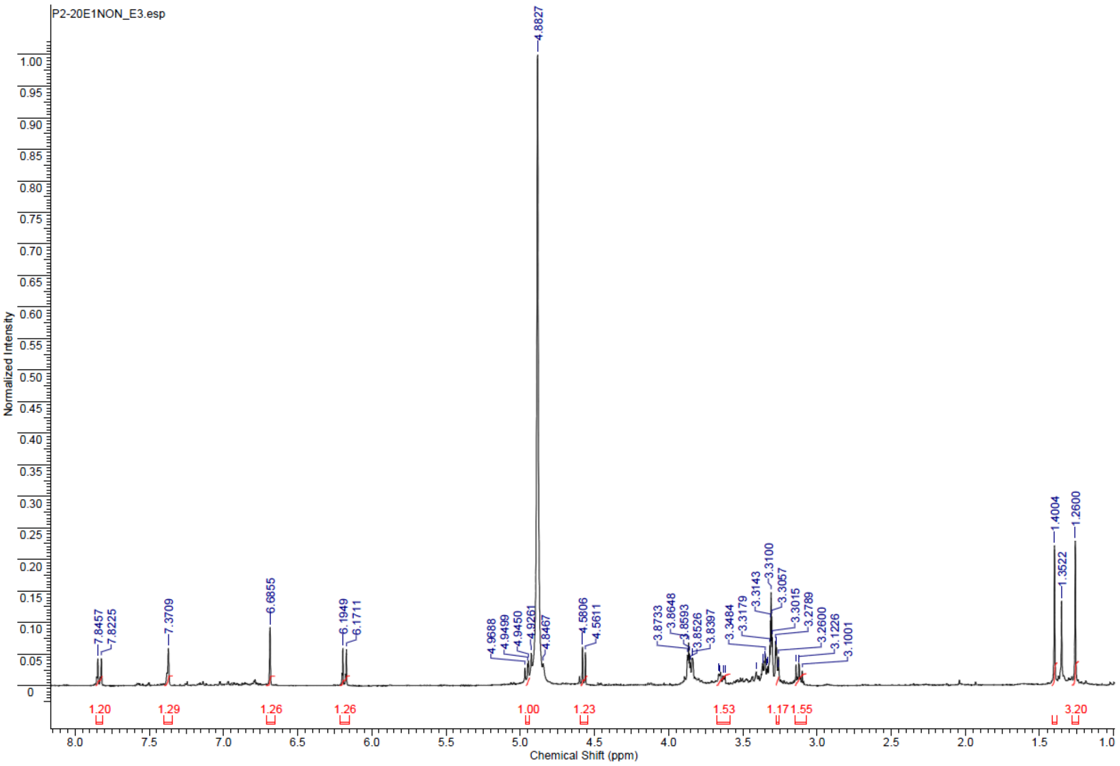

Figure S4. ^{1}H-NMR spectra of (-)-marmesinin (**2**).

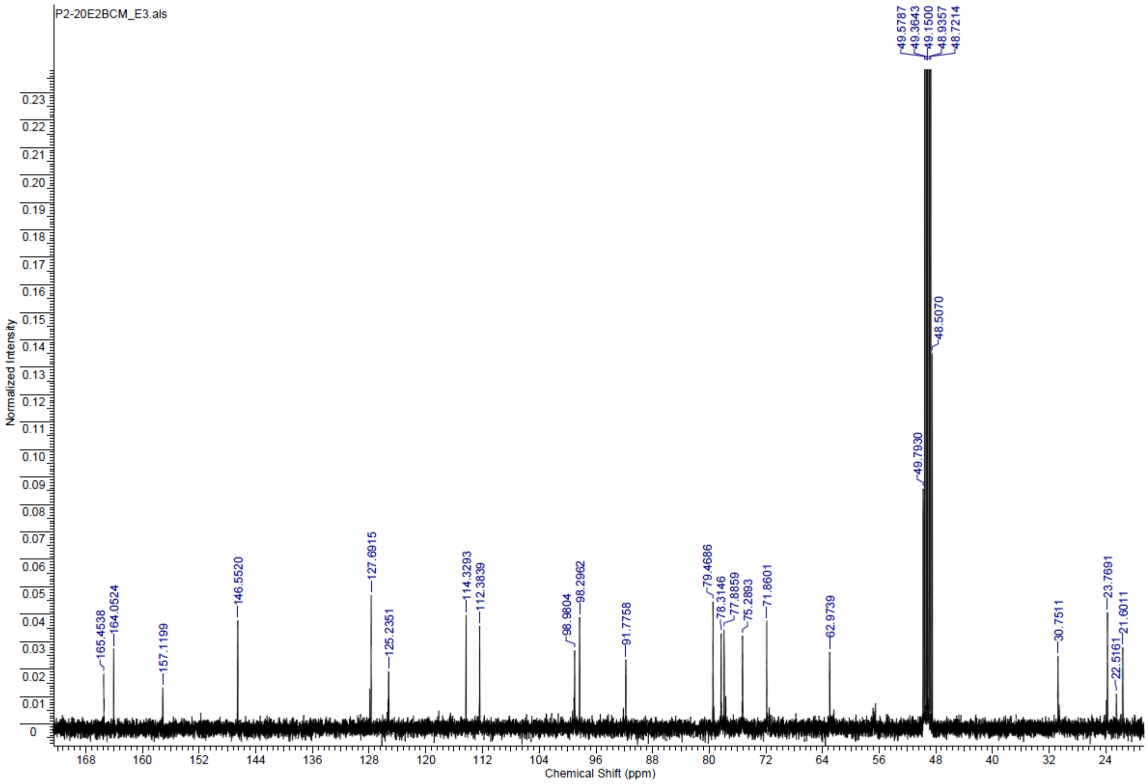

Figure S5. ^{13}C-NMR spectra of (-)-marmesinin (**2**).

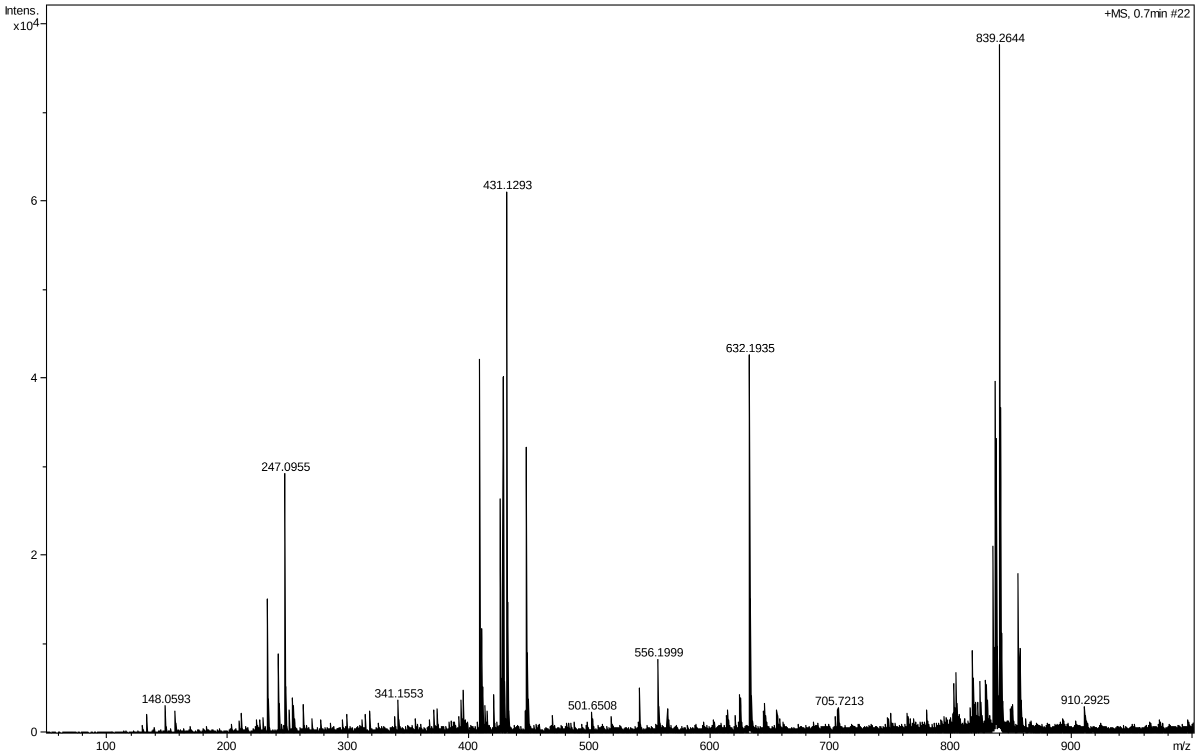

Figure S6. ESI-MS spectra of (-)-marmesinin (**2**).

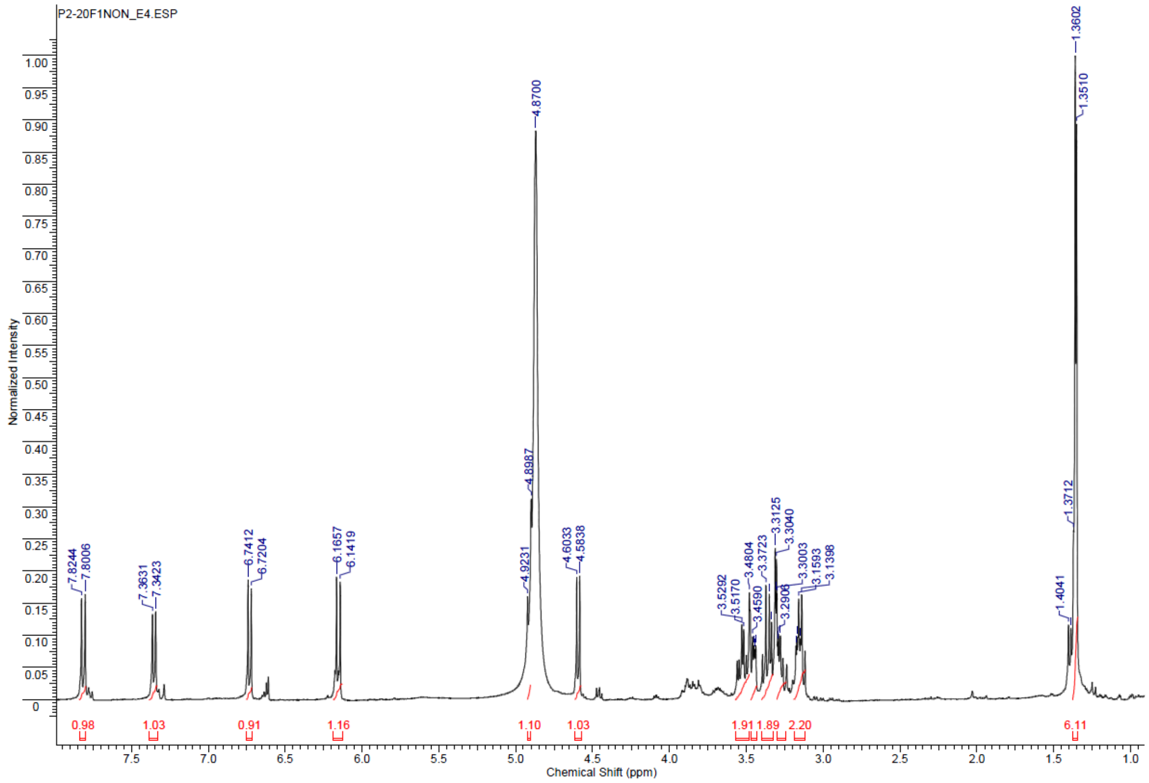

Figure S7. ^{1}H-NMR spectra of columbianetin β-D-glucopyranoside (**3**).

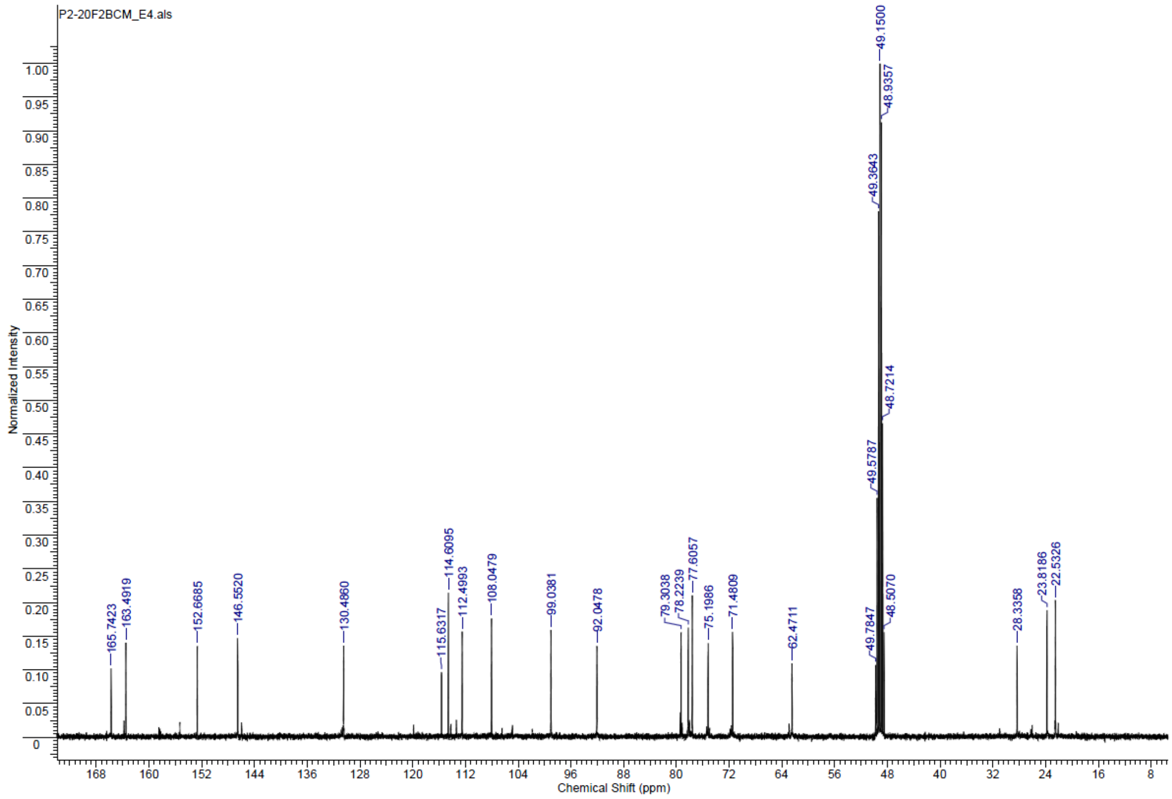

Figure S8. ^{13}C-NMR spectra of columbianetin β-D-glucopyranoside (**3**).

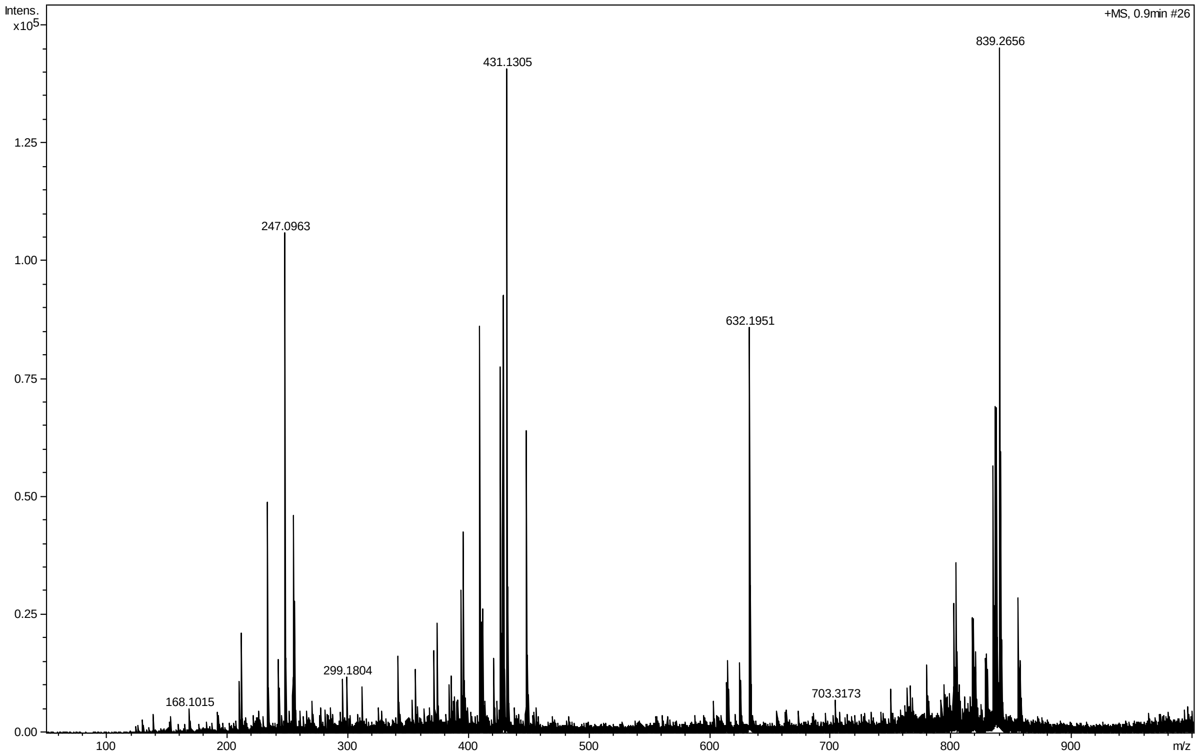

Figure S9. ESI-MS spectra of columbianetin β-D-glucopyranoside (**3**).

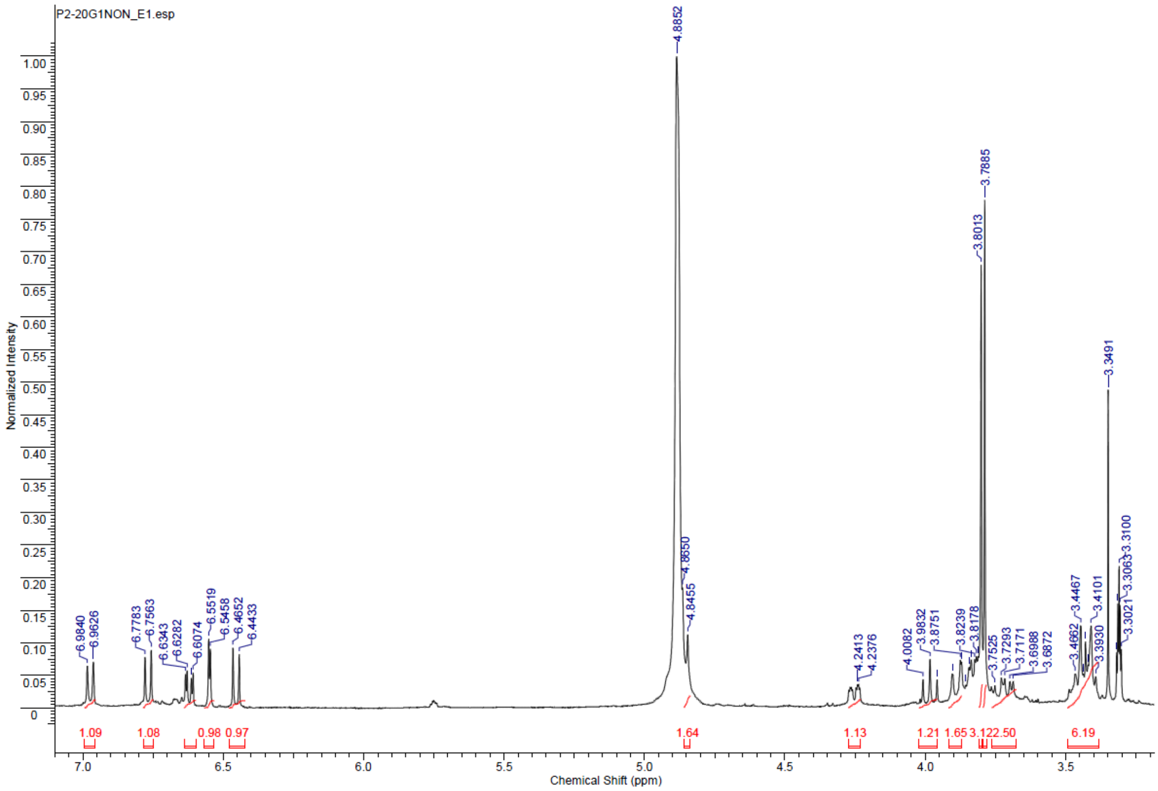

Figure S10. ^{1}H-NMR spectra of], isomucronulatol 7-*O*-β-D-glucoside (**4**)

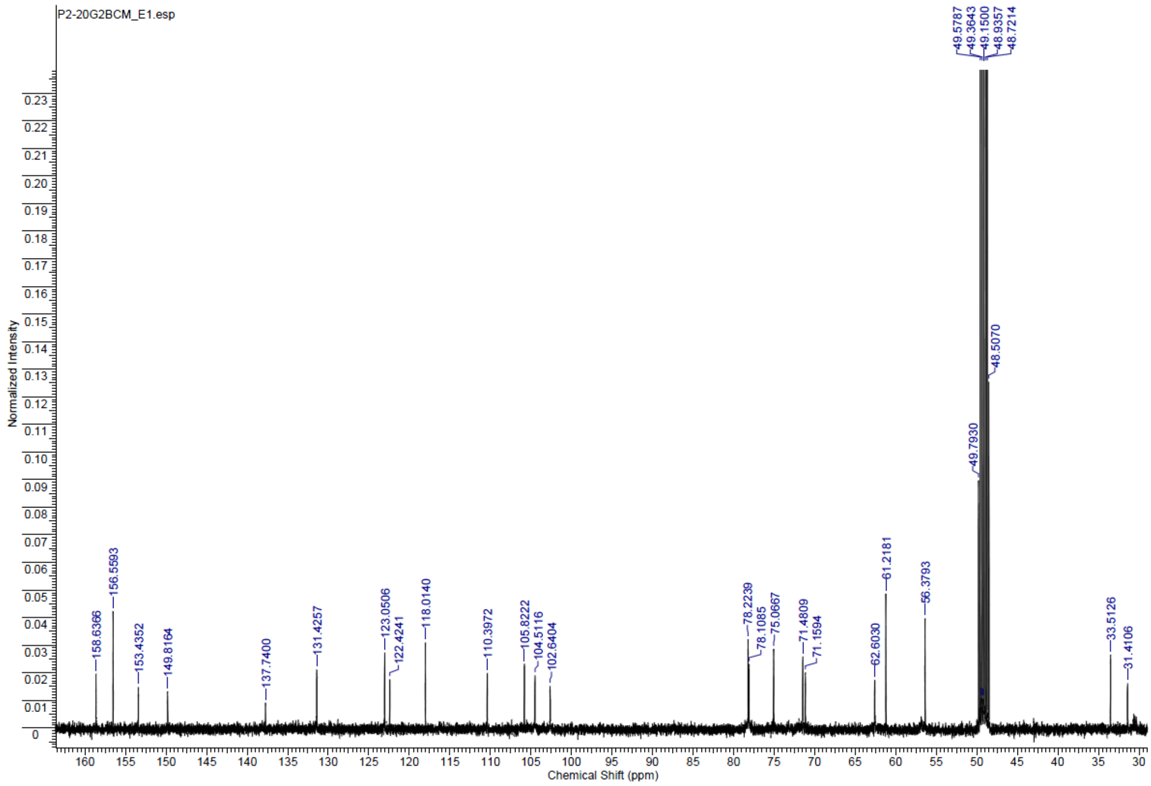

Figure S11. ^{13}C-NMR spectra of isomucronulatol 7-*O*-β-D-glucoside (**4**).

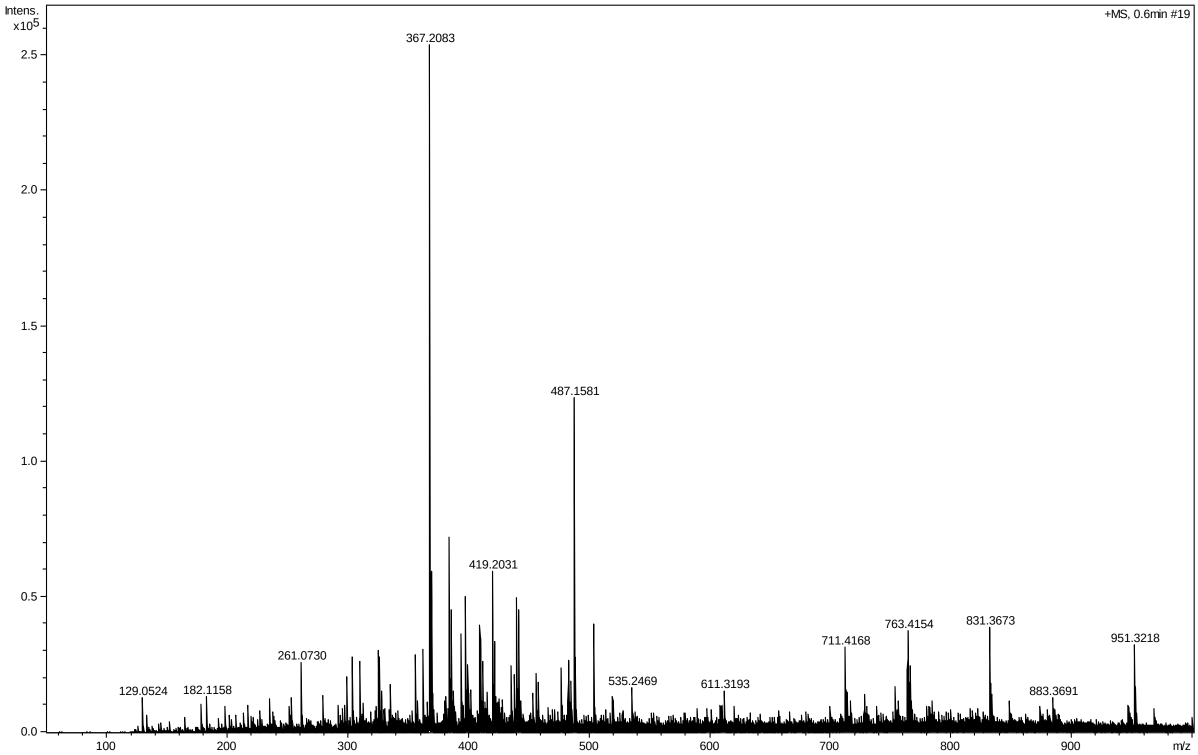

Figure S12. ESI-MS spectra of isomucronulatol 7-*O*-β-D-glucoside (**4**).

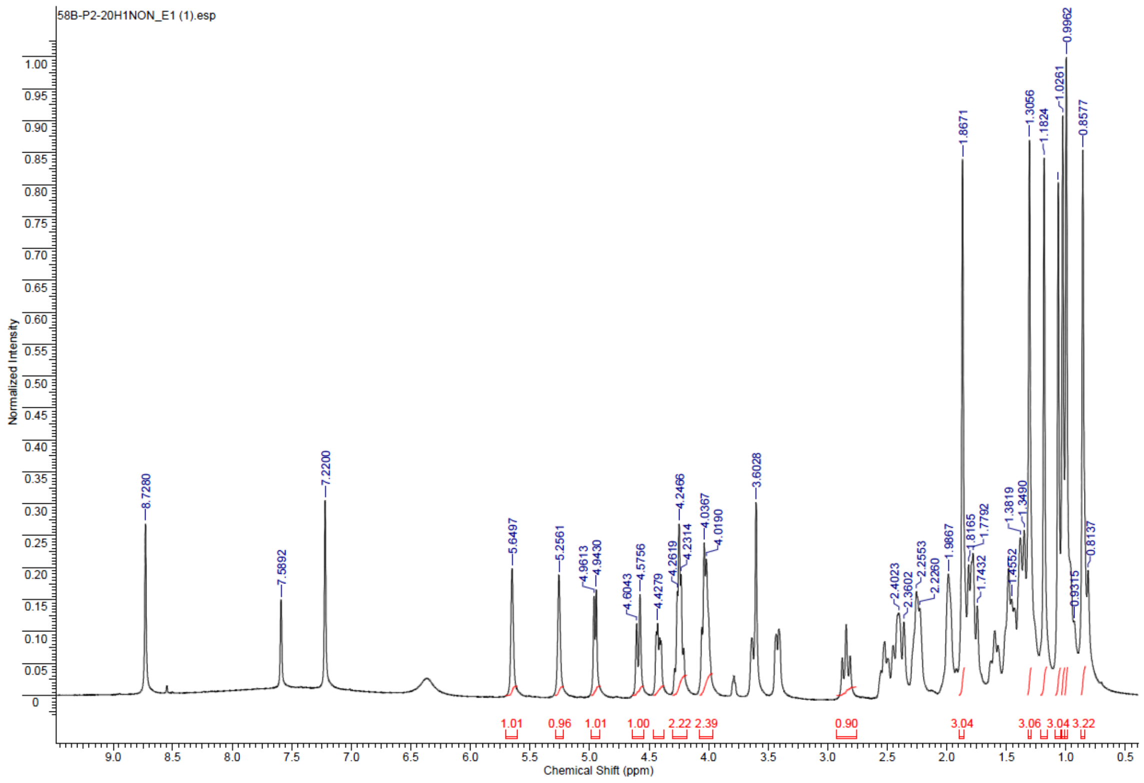

Figure S13. ^{1}H-NMR spectra of ecliptasaponin A (**5**).

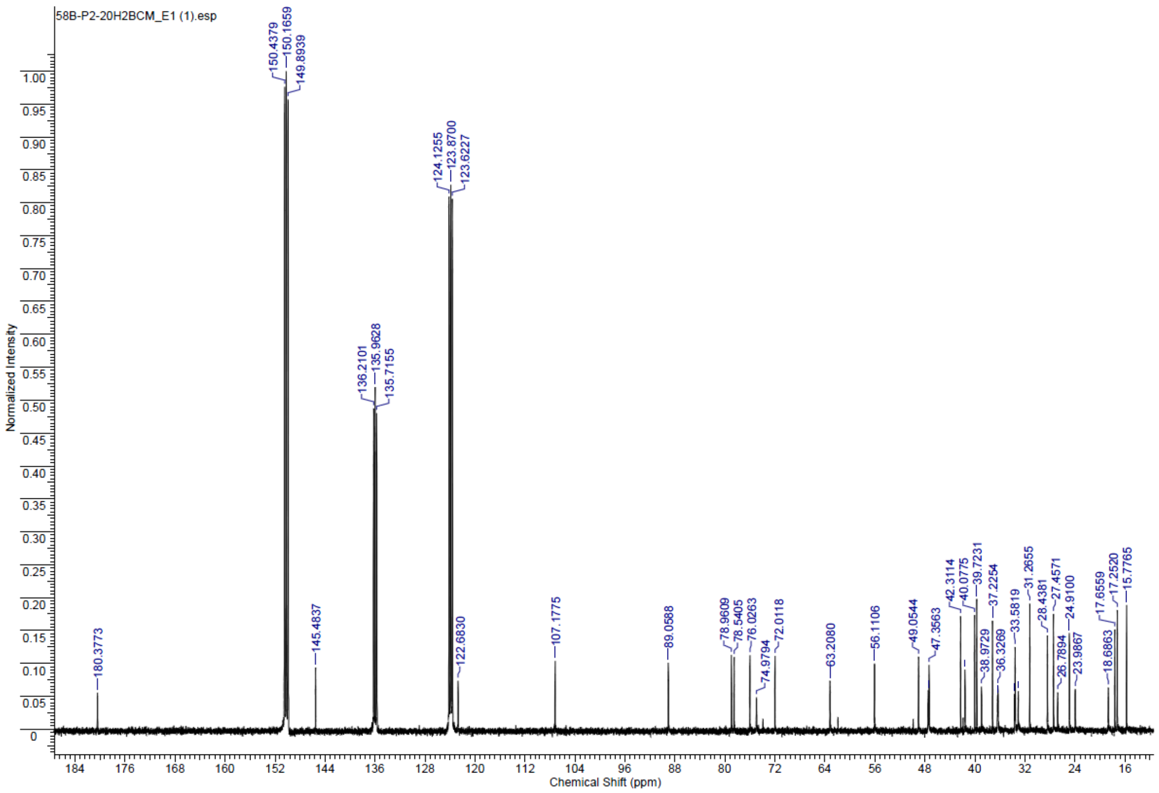

Figure S14. ^{13}C-NMR spectra of ecliptasaponin A (**5**).

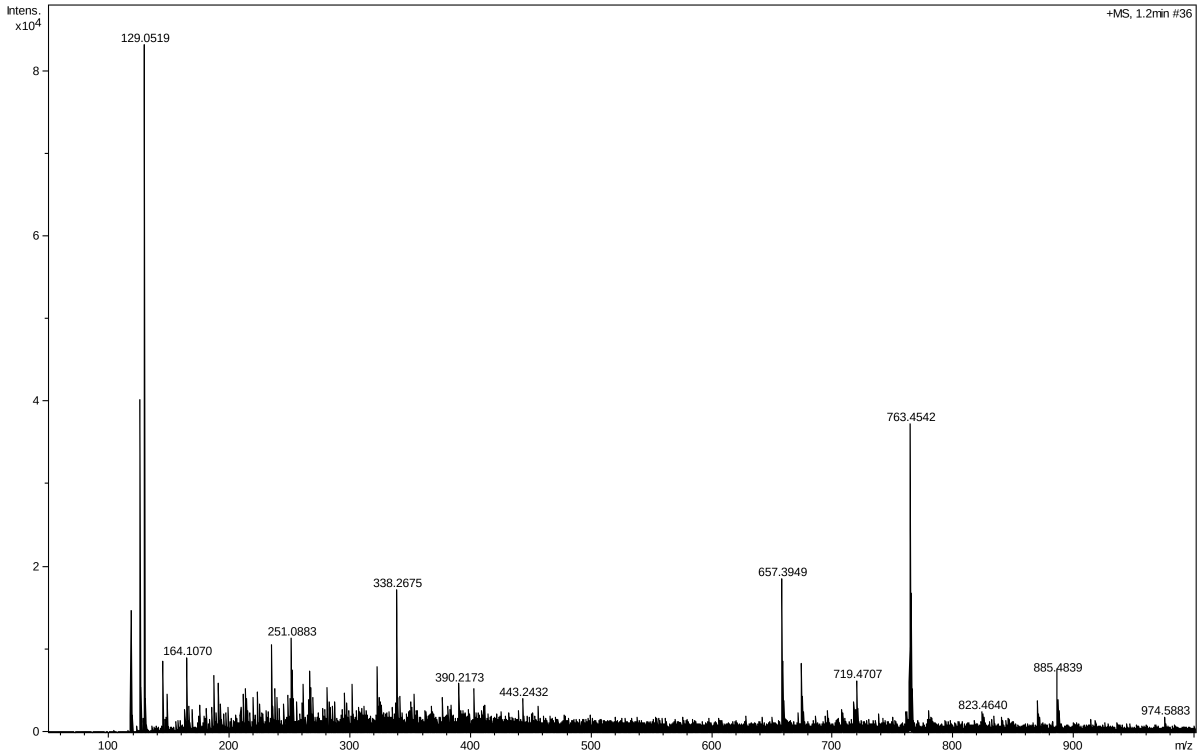

Figure S15. ESI-MS spectra of ecliptasaponin A (**5**).
